# Effect of Api-Bioxal^®^ and ApiHerb^®^ Treatments against *Nosema ceranae* Infection in *Apis mellifera* Investigated by Two qPCR Methods

**DOI:** 10.3390/vetsci7030125

**Published:** 2020-09-04

**Authors:** Giovanni Cilia, Claudia Garrido, Martina Bonetto, Donato Tesoriero, Antonio Nanetti

**Affiliations:** 1 Department of Veterinary Sciences, University of Pisa, Viale delle Piagge 2, 56124 Pisa, Italy; giovanni.cilia@vet.unipi.it; 2 CREA Research Centre for Agriculture and Environment, Via di Saliceto 80, 40128 Bologna, Italy; martina.bonetto@outlook.it (M.B.); donato.tesoriero@crea.gov.it (D.T.); antonio.nanetti@crea.gov.it (A.N.); 3 BeeSafe-Bee Health Consulting for Veterinary Medicine and Agriculture, 59071 Hamm, Germany

**Keywords:** microsporidia, *Nosema ceranae*, *Hsp70* gene, *16S rRNA* gene, oxalic acid, garlic

## Abstract

*Nosema ceranae* is a worldwide distributed midgut parasite of western honey bees, leading to dwindling colonies and their collapse. As a treatment, only fumagillin is available, causing issues like resistance and hampered bee physiology. This study aimed to evaluate ApiHerb^®^ and Api-Bioxal^®^ as treatments against *N. ceranae*. The efficacy was tested using two qPCR methods based on the *16S rRNA* and *Hsp70* genes. In addition, these methods were compared for their aptitude for the quantification of the infection. For this, 19 colonies were selected based on the presence of *N. ceranae* infections. The colonies were divided into three groups: treated with ApiHerb, Api-Bioxal with previous queen caging and an untreated control. All colonies were sampled pre- and post-treatment. The bees were analyzed individually and in duplicate with both qPCR methods. All bees in the pre-treatment tested positive for *N. ceranae*. Both treatments reduced the abundance of *N. ceranae*, but ApiHerb also decreased the prevalence of infected bees. Analysis with the *16S rRNA* method resulted in several orders of magnitude more copies than analysis with the *Hsp70* method. We conclude that both products are suitable candidates for *N. ceranae* treatment. From our analysis, the qPCR method based on the *Hsp70* gene results as more apt for the exact quantification of *N. ceranae* as is needed for the development of veterinary medicinal products.

## 1. Introduction

Nosemosis Type C is a worldwide occurring disease of western honey bees (*Apis mellifera*) caused by *Nosema ceranae* [1]. This microsporidium was identified in the Asian honey bee *Apis cerana* [2], which is generally considered the original host [3]. In recent decades, the prevalence of this parasite highly increased also in western honey bees, causing the colonies to decline and collapse [4,5].

*N. ceranae* is an intracellular obligate parasite and infects the epithelial cells of the ventriculum [2,6] with high tropism for this organ [7]. Nosemosis Type C shows symptoms both at the individual and colony level, including lifespan reduction, lethargic behavior and poor honey and pollen harvest [5,8,9]. Moreover, in some cases, *N. ceranae* infections tend to be asymptomatic, with features difficult to spot in the field [2,10,11].

Besides *A. cerana* and *A. mellifera*, the microsporidium was reported in several other hymenopteran species, and was also found in regurgitated pellets of bee-eaters [12,13,14,15,16]. It was also found in small hive beetles (*Aethina tumida)* [17], although the possibility of transmission to hymenopterans remains unclear [18].

In addition, the pathogen is transmitted during common flower visits to other bee species [19,20]. This gives finding efficient treatments an added, ecological importance: the emergent disease could pose a threat to wild pollinators and to the stability of pollination services. To avoid further dispersal, an efficient control in managed bee populations is crucial. So far, the only effective treatment against nosemosis is fumagillin, a mycotoxin derived from *Aspergillus fumigatus* [21,22]. However, fumagillin treatment could contribute to the development of resistant *N. ceranae* strains and stable residues in honey. Further, being toxic to mammals, treatments with this compound may create issues for food safety. Fumagillin treatments may also affect bee physiology and promote parasite development [23]. Finally, this compound is not legally available in all countries.

Therefore, finding treatments alternative to fumagillin is of high importance for honey bee health as well as for avoiding ecological issues. Recently, some formulations with natural compounds have been evaluated for the treatment of Nosemosis Type C in honey bee colonies [24,25,26]. ApiHerb^®^, a commercial dietary supplement, showed effects against *N. ceranae* infections [27,28]. Similarly, oxalic acid, an organic acid used for treatments against the parasitic mite *Varroa destructor* [29,30,31], was found to be efficient against *N. ceranae* both in the laboratory and the field [32,33]. In addition, oxalic acid treatments are usually done in broodless conditions. In summer, this is achieved by caging the queen [34], but brood interruptions consequent to natural requeening have been shown to be beneficial against *N. ceranae* infections [35]. 

The aim of this study was to comparatively evaluate the effect of the dietary supplement ApiHerb^®^, and Api-Bioxal^®^, a registered veterinary drug against *Varroa destructor* based on oxalic acid dihydrate. We assessed the efficacy of these two treatments on *N. ceranae* infections under field conditions. 

To measure the efficacy of treatments, exact quantification of the *Nosema* infection is crucial. Therefore, we compared two available qPCR methods, respectively based on the *16S rRNA* and *Hsp70* genes. The aim of this comparison was to gain insight into the aptitude of these methods for the quantification of the infection.

## 2. Materials and Methods

### 2.1. Experimental Design

The experiment was made in autumn 2017, in an apiary of CREA—Research Centre for Agriculture and Environment located in Bologna, Italy (44°31′27.1″ N 11°21′03.6″ E). The apiary consisted of approximately forty *Apis mellifera ligustica* colonies housed in ten-frame Dadant-Blatt hives.

Nineteen of those colonies were selected for the experiment based on the presence of *N. ceranae* infection, which was detected in a preliminary screening made on pooled samples of 25 bees collected from the external combs of each colony, and analyzed with the *Hsp70* qPCR method (see Section 2.2). Those colonies were then randomly divided into the three treatment groups AB (N = 7), AH (N = 6) and C (N = 6).

All treatments were made with sugar water (1:1, *w*/*w*) that was added with the underreported formulations following the label instructions and then trickled once or three times with a syringe onto the combs at the dose of 50 mL, as described on the label. In the colonies of Group AB, the queens were kept in queen-excluder cages (Var-Control, API-MO.BRU, Padua, Italy) for 21 days (15/9–6/10) to prevent egg laying. At the end of the period, the queens were released and the broodless colonies received an Api-Bioxal (Chemicals Laif SpA, Padua, Italy) solution. The colonies of Group AH were unmanipulated, broodright and with a laying queen. They were treated three times with an ApiHerb (Chemicals Laif SpA, Padua, Italy) solution, at one-week intervals (6, 13 and 20/10). The above treatments correspond to the posology indicated by the manufacturer. The colonies of Group C served as negative controls and were left unmanipulated and untreated. 

**Figure 1:**
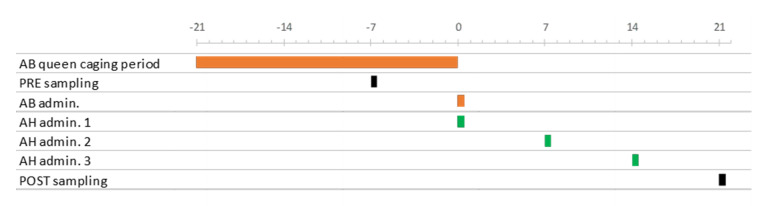
Summary of the working schedule. The horizontal axis represents the timeline, with day 0 corresponding to the 6 October 2017. AB (orange) and AH (green) indicate respectively the Api-Bioxal- and ApiHerb-treated groups.

Twenty-five adult honey bees were sampled pre-treatment (15/9) and post-treatment (27/10) from the external combs of each colony and stored at −20 °C until analysis (Figure 1).

### 2.2. DNA Extraction and qPCR Analysis

Each sampled honey bee was analyzed individually after careful dissection. The digestive tract from the ventriculum to the rectum was removed with tweezers and homogenized in 1 mL DNAse-free water with Tissue Lyser II (Qiagen, Hilden, Germany) for 3 min at 30 Hz.

The total DNA was extracted from each homogenate with a Quick DNA Microprep Plus Kit (Zymo Research, Irvine, CA, USA) following the manufacturer’s instructions for solid tissue processing.

Two aliquots from each DNA extract were taken and analyzed separately in duplicate by qPCR with primers and probes specific for *N. ceranae*, and respectively designed on sequences of the *16S rRNA* [36] and *Hsp70* [37] genes (Table 1). For each target gene, a total reaction volume of 15 μL was prepared using 2x QuantiTect Probe PCR Master Mix (Qiagen, Hilden, Germany), forward and reverse primers (2 μM), forward and reverse probes (500 nM) and 3 μL DNA extract. 

Purified *N. ceranae*-specific amplicons were individually incorporated into a cloning vector using the TA Cloning™ Kit with the pCR™2.1 Vector (Invitrogen, Carlsbad, CA, USA), following the manufacturer’s instructions. Recombinant plasmid DNA was purified using the Plasmid Mini Prep Kit (BIO-RAD, Hercules, CA, USA). The copy number of plasmid DNA was calculated based on the molar concentration and molecular mass of the recombinant plasmid consisting of the plasmid vector and the PCR insert. Each template of recombinant plasmids containing the *N. ceranae*-specific DNA fragment was diluted to 10^0^ to 10^9^ copies. The standard curve was generated by amplifying the serially diluted plasmidas in a duplex qPCR assay. The real-time PCR assay was performed on a Rotorgene Corbett 6000 (Corbett Research, Sydney, Australia) following the amplification and quantification protocols for either gene sequence [36,37]. All the analyses were conducted with two technical replicates for each target gene.

### 2.3. Statistical Analysis

The *N. ceranae* abundance was determined at the individual bee level (N = 950) by averaging the two technical replicates of each PCR method. Initially, the individual data were tested against the independent categorical variable “colony” to detect intra-apiary differences. In a second step, the individual bees were also considered technical replicates needed to assess the abundance at the colony level. A new database was then created with the average number of copies detected in the colonies with either method at both sampling points. This database was used to calculate the corresponding prevalence data (i.e., the proportion of positive individuals in the samples (N = 25)) and the relative pre–post variation ($\mathrm{RV}=\frac{\mathrm{POST}-\mathrm{PRE}}{\mathrm{PRE}}$) of both abundance and prevalence, that were expressed as percentages. The variables above were tested against the “treatment” as a categorical factor.

Extensive violations to the assumptions of normality and homogeneity of variance were found respectively with the Shapiro–Wilks and Levene’s tests. Significant violation of normality remained even after the application of the angular transformation of proportions ($x_{1}=\sin^{-1}\sqrt{x}$) and the log transformation of the other continuous variables ($x_{1}=\log x+1$), which made it not possible to analyze the data with a parametric approach.

The effect of categorical factors was therefore tested non-parametrically, by a Kruskal–Wallis one-way ANOVA for independent samples and, when needed, with a post hoc test for multiple comparisons with Bonferroni’s correction.

The number of *N. ceranae* copies detected in the same individual honey bee with the two qPCR methods was compared with a two-tailed paired-samples *T* test. Due to the large sample size, checking for the normality assumption was not considered stringent in this case.

The association between continuous variables was evaluated with a two-tailed Pearson’s correlation. Besides, the abundance values obtained with both methods for each sampled honey bee were divided (*16S rRNA*/*Hsp70*) and the ratio was used as the dependent variable in a linear regression model with the *Hsp70* abundance as the explanatory independent variable. Cases generating a divide-by-zero error were excluded, which resulted in the analysis being conducted on a subsample of the original dataset (N = 875).

Frequencies were compared with the Pearson’s χ^2^ test for independence.

## 3. Results

### 3.1. N. ceranae Infection in Test Colonies

All the forager bees collected pre-treatment generated a PCR signal for *N. ceranae* with both methods, resulting in 100% prevalence in all colonies.

The *N. ceranae* abundance measured with the *16S rRNA* method averaged 1.50 +/− 0.34 s.e. (SD = 7.48, 95% CI = 0.82, 2.17) × 10^12^ copies per bee. When the same samples were analyzed with the *Hsp70* method, a lower average abundance value of 3.31 +/− 0.66 s.e. (SD = 14.39, 95% CI = 2.01, 4.60) × 10^9^ *Nosema* copies per bee was measured (t(474) = 4.354, *p* = 0.000) (Table 2).

In both cases, the *Nosema* abundance at the individual bee level was influenced by the colony (*16S rRNA*: H(18) = 468.26, *p* = 0.000; *Hsp70*: H(18) = 456.21, *p* = 0.000), but not by the treatment group (*16S rRNA*: (H(2) = 0.031, *p* = 0.985; *Hsp70*: H(2) = 0.277, *p* = 0.870).

### 3.2. Treatment Effect

The *N. ceranae* abundance in the post-treatment samples averaged 2.60 +/− 0.51 s.e. (SD = 11.11, 95% CI = 1.60, 3.60) × 10^7^ and 4.82 +/− 0.86 s.e. (SD = 18.71, 95% CI = 3.13, 6.51) × 10^5^ copies per bee, respectively with the *16S rRNA* and *Hsp70* methods. Further, in this case, the second method resulted in lower abundance values (t(474) = 5.082, *p* = 0.000).

The number of *N. ceranae* copies detected with either method was influenced by both colony (*16S rRNA*: H(18) = 459.02, *p* = 0.000; *Hsp70*: (H(18) = 458.69, *p* = 0.000) and treatment group (*16S rRNA*: H(2) = 14.20, *p* = 0.001; *Hsp70* (H(2) = 13.66, *p* = 0.001) (Table 3). In detail, a multiple comparison test showed differences between AH and C (with either method: *p* = 0.001) and AB and C (*16S rRNA*: *p* = 0.064; *Hsp70*: *p* = 0.049), but not between AH and AB (*16S rRNA*: *p* = 0.497; *Hsp70*: *p* = 0.341).

The treatments also influenced the relative variation of prevalence (*16S rRNA*: H(2) = 7.25, *p* = 0.027; *Hsp70*: (H(2) = 7.31, *p* = 0.026). In the AB and C groups, the prevalence remained at 100%, i.e., the pre-treatment level. On the other hand, in AH colonies, it decreased by 45.33% +/− 20.39 s.e. (SD = 49.94, 95% CI = −97.74, +7.07) and 50.00% +/− 22.36 s.e. (SD = 54.77, 95% CI = −107.48, +7.48) when determined with *16S rRNA* and *Hsp70*, respectively (Table 4).

The percent variation of abundance was also influenced by the treatments (*16S rRNA*: (H(2) = 13.90, *p* = 0.001; *Hsp70*: (H(2) = 14.92, *p* = 0.001) (Table 5). 

The controls differed from both Groups AH (*16S rRNA*: *p* = 0.001; *Hsp70*: *p* = 0.000) and AB (*16S rRNA*: *p* = 0.056; *Hsp70*: *p* = 0.080), but no difference could be detected between the treated groups (*16S rRNA*: *p* = 0.4141; *Hsp70*: *p* = 0.224).

### 3.3. Comparability of the 16S rRNA and Hsp70 Methods

A paired-samples *T* test conducted on the pre- and post-treatment bee samples resulted in a higher number of copies detected with the *16S rRNA* (7.48 +/− 1.73 s.e., SD = 53.44 × 10^11^) compared to the *Hsp70* method (1.65 +/− 0.33 s.e., SD = 10.31 × 10^9^) (t(949)= −4.314, *p*= 0.000). A positive correlation could be calculated between the two series of analytical data (r(950) = +0.859, *p* = 0.000).

In a linear regression model, the *16S rRNA*/*Hsp70* ratio was considered the dependent variable against the *Hsp70* abundance as the independent predictor (Table 6).

**Figure 2:**
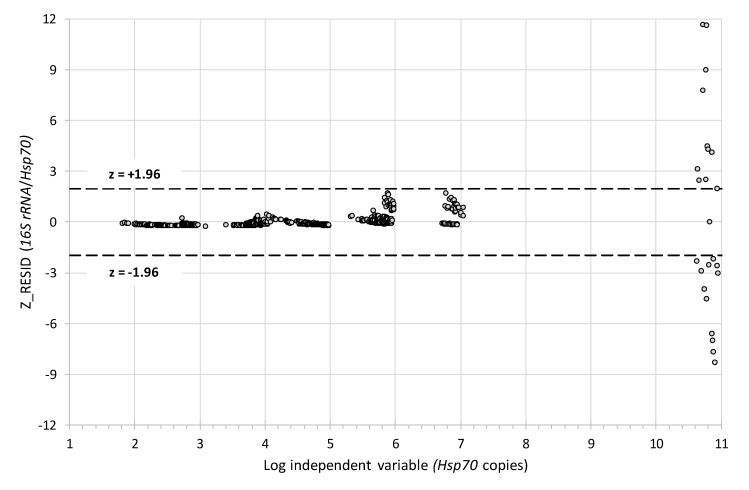
Scatterplot showing the relationship between the error variance of the *16S rRNA*/*Hsp70* ratio and the abundance measured with the *Hsp70* method (N = 875). The interval of the 95% normally distributed data (z = +/− 1.96) is shown as a reference.

Despite that the model and both regression parameters could be confirmed (*p* = 0.000), the analysis of the standardized regression residuals showed a pattern of progressive spread as the independent variable increased (Figure 2).

## 4. Discussion

This trial was conducted to address the largely understudied problem of *N. ceranae* infection management in contexts where the use of antibiotics is ruled out, raising the consequent demand of naturally based treatments.

The colonies included in the experiment were apparently healthy, of regular development for the considered area and season, and did not show signs of dwindling. Despite the good general colony conditions, all the old workers that were sampled pre-treatment from the external combs were found infected. The genetic copies found in the bee intestines with the two q-PCR techniques varied deeply in number between the colonies. The detected high prevalence, compared to previous knowledge on the disease obtained in a similar southern European environment [5], makes it likely that the experimental colonies were approaching the collapse threshold. This may be particularly true for the colony 3AB, which showed conspicuous *N. ceranae* abundance in all sampled bees. It is worth mentioning that the high average abundance detected with both methods makes this colony a putative strong outlier. However, as that was a normal, naturally infected colony of the apiary, its removal from the dataset was considered arbitrary and, for the information content coming from this highly infected and nevertheless asymptomatic case, unjustified also.

The products under consideration were ApiHerb, a patented herbal feeding supplement for honey bees, and Api-Bioxal, a formulation containing dihydrate oxalic acid as the active ingredient. The latter is registered in various countries to control varroa infestations. Previous trials confirmed an influence of either product on the midgut microbiota of honey bees fed in laboratory conditions [38].

The quantitative ApiHerb composition is patent-protected and, therefore, it has not been disclosed. However, the label reports, in decreasing order, dextrose, garlic (*Allium sativum*) and cinnamon (*Cinnamomum zeylanicum*) as the three most abundant ingredients, plus a range of vitamins and flavors. Excluding dextrose as a possible active ingredient and considering that non-confidential information from the manufacturer reports garlic and cinnamon to be respectively contained within the ranges 25–50 and 1–5% [personal communication], it is likely that garlic is the major ingredient responsible for the ApiHerb effect detected on the intestinal microflora of the honey bees.

Garlic preparations and components are reported to have antifungal properties [39,40,41,42]. In in vitro tests, aqueous extracts of this plant resulted in decreased spore vitality in *N. bombycis*, the agent of the pebrine disease of silkworm, and, once administered in vivo to *Bombyx mori* larvae, in a reduced prevalence of infected individuals [43]. When administered in the laboratory as ethanolic extracts to artificially *N. ceranae*-infected honey bees, the treatment did not result in significant infection inhibition, as a possible consequence of the low allicin stability in the solvent that was used [44]. However, the administration in sugar water after solubilization into ethanol of allyl sulphide, another garlic compound, significantly reduced the abundance of *N. ceranae* spores in artificially infected bees [45].

Previous trials highlighted the ability of ApiHerb to inhibit *N. ceranae* infections in *A. mellifera*. In a laboratory assay, caged bees were fed ApiHerb for 24 h. Afterwards, they were infected and fed sugar water for ten days, until dissection. In comparison to untreated controls, they showed significantly less *N. ceranae* spores [46]. Field tests resulted in a significant decrease in both number of spores (Italy and Mexico) and prevalence of infected house and forager bees (Spain) compared to untreated controls [47,48].

In the present trial, after four ApiHerb administrations, the post-treatment abundance was various orders of magnitude lower than the pre-treatment level, corresponding to a variation approaching −100%. This drastic and slightly variable effect must be compared with the high and nevertheless very variable increased abundance recorded in the untreated controls in the pre-/post-treatment period. The experiment allowed us to measure also the proportion of infected individuals on the same sample bees. In ApiHerb-treated colonies, the prevalence of infected bees decreased by approximately 45–50%, with variations depending on the q-PCR method that was used, resulting in some negative colonies at the post-treatment check.

The potential of natural queen replacement and the consequent brood hiatus in the control of *N. ceranae* infections was pointed out previously [35], although the dynamics of the healing effect could not be fully clarified. In an independent experiment, a 0.25 M oxalic acid solution was administered twice by trickling to naturally infected free-flying colonies, and by feeding to artificially infected honey bees reared in the laboratory, with an effect on prevalence and abundance, respectively [29]. In this trial, the Api-Bioxal treatment corresponded to a single administration of a 0.47 M oxalic acid solution, that was trickled as a single dose to colonies that were artificially broodless after caging the queen. In a way, this treatment combined two techniques—artificial brood interruption and oxalic acid administration—that showed an effect against the infection in separate experiments. Therefore, it is likely that the recorded effect is the result of the combination between two independent actions.

In the pre-/post-treatment interval, the abundance decreased by one or more orders of magnitude in all Api-Bioxal-treated colonies, corresponding to an approximate average variation of −99%. Although this difference did not diverge significantly from the one recorded in the ApiHerb-treated colonies, it must be noted that the Api-Bioxal treatment did not result in a reduced prevalence also. This contrasts with the above-mentioned experiment, where repeated administrations may have resulted in prolonged coverage and a consequent effect on the prevalence of infected individuals. 

Except for the season of application, the Api-Bioxal treatment of this trial simulates the routine varroa control technique used by the beekeepers treating their colonies with oxalic acid after a period of queen caging [34], therefore possibly acting as a double-effect treatment.

The two assays in this study are based on different qPCR targets, *16S rRNA* and *Hsp70*, and characterized by high repeatability [36,37,49,50]. However, there were systematic differences between the two methods. The assay based on the *16S rRNA* gene measured a higher number of copies than those detected with the *Hsp70* gene. The ratio between the measurements obtained with the two methods is not constant. The significant, positive slope resulting from the regression analysis showed that the results of the *16S rRNA* analysis diverge significantly with an increasing number of copies detected with the *Hsp70* method. The residue analysis shown in Figure 2 indicates an uneven distribution of the error variance throughout the number of copies resulting from the analysis using the *Hsp70* gene and, therefore, problematic use of such a model in the straightforward conversion of the abundance values between the two methods.

Both methods have the same linear dynamic range (LDR) and efficiency [36,37]. The diverging results, therefore, arise not because of sensitivity issues, but the different properties of the compared methods. *16S rRNA* is a multi-copy gene and it is present in microsporidia with a variable number of sequences [51]. The fluctuations in the number of copies of the *16S rRNA* gene in the genome of *N. ceranae* may ultimately affect the quantification [52]. In addition, studies on *N. bombi* showed that genes coding for rRNA were intrinsically polymorphic and of elusive nature [53]. These results suggest that high caution is necessary for using these microsporidian sequences, especially when quantification is needed.

On the other hand, *Hsp70* is a single-coding gene in a highly conserved region of the *N. ceranae* genome, which results in accurate quantification [54,55]. In the development of veterinary medicines against Nosemosis Type C, a precise evaluation of both abundance and prevalence reduction is required, which makes the use of quantification methods based on single-copy gene sequences essential. The method based on the *Hsp70* gene, therefore, seems more appropriate for the evaluation of efficacy against *N. ceranae*. 

## 5. Conclusions

Despite the wide-spread use of fumagillin in many places, we demonstrate that it is possible to efficiently control an emergent pathogen like *N. ceranae* with sustainable methods. The products used in this study, however, were not developed for the treatment of *N. ceranae*. For an extension of use, further research is necessary. In a first step, a scientifically based optimization of parameters like the concentration of active substances, treatment period, and posology is still needed. Studies are needed to elucidate the mode of action against *N. ceranae* of oxalic acid as well as the compounds in ApiHerb.

Given the increasing importance of Nosemosis Type C worldwide, safe veterinary medicinal products to control this disease become urgent. In this context, standardizing the protocols with a guideline for the development of these products is an evident requirement. Until now, such a guideline only exists for the registration of treatments against the parasitic mite *Varroa destructor* [56]. With honey bee health becoming an increasingly serious issue for food security, extending these standards to further honey bee diseases appears an indispensable adaptation. Future research focusing on developing integrated treatments will provide us with a more holistic view of honey bee health.

## Figures and Tables

**Table 1:** Primer sequences used for the qPCR analysis with the *Hsp70* and *16S rRNA* genes and TaqMan Probes.

Gene	Primers	Sequence (5′–3′)	Reference
*16S rRNA*	Forward	AAGAGTGAGACCTATCAGCTAGTTG	[36]
	Reverse	CCGTCTCTCAGGCTCCTTCTC	
	TaqMan Probe	ACCGTTACCCGTCACAGCCTTGTT	
*Hsp70*	Forward	GGGATTACAAGTGCTTAGAGTGATT	[37]
	Reverse	TGTCAAGCCCATAAGCAAGTG	
	TaqMan Probe	TGAGCCTACTGCGGC	

**Table 2:** Mean abundance of *N.* *ceranae* copies detected with the two qPCR methods in the samples collected pre-treatment from the experimental colonies. As data covered various orders of magnitude, for ease of reading, logarithmic representation was adopted. Standard error of means (s.e.) and standard deviations (SD) are shown also.

			*16S rRNA*		*Hsp70*	
Colony	Group	N	Mean +/− s.e.	SD	Mean +/− s.e.	SD
3	AB	25	13.45 +/− 12.55	13.25	10.80 +/− 9.45	10.15
4	AB	25	6.73 +/− 5.00	5.70	5.72 +/− 4.24	4.94
5	AB	25	4.73 +/− 3.15	3.85	3.86 +/− 2.23	2.93
8	AB	25	4.75 +/− 3.09	3.79	3.86 +/− 2.22	2.92
10	AB	25	7.75 +/− 6.26	6.96	5.92 +/− 4.22	4.92
13	AB	25	7.09 +/− 5.85	6.55	5.85 +/− 4.30	4.99
14	AB	25	4.54 +/− 3.06	3.76	3.84 +/− 2.39	3.09
F1	AH	25	7.13 +/− 5.67	6.36	5.84 +/− 4.39	5.09
F3	AH	25	7.17 +/− 5.88	6.58	5.79 +/− 4.29	4.99
16	AH	25	5.51 +/− 3.93	4.63	4.30 +/− 3.23	3.93
22	AH	25	6.77 +/− 5.15	5.85	5.58 +/− 4.24	4.94
24	AH	25	5.20 +/− 3.84	4.53	3.93 +/− 2.40	3.10
25	AH	25	4.14 +/− 3.31	4.01	3.70 +/− 2.40	3.10
F4	C	25	5.68 +/− 4.07	4.77	4.68 +/− 3.35	4.05
F7	C	25	5.43 +/− 3.90	4.60	4.66 +/− 3.29	3.99
F9	C	25	4.90 +/− 3.00	3.70	3.91 +/− 2.43	3.12
17	C	25	5.57 +/− 3.92	4.62	4.78 +/− 3.07	3.77
19	C	25	6.94 +/− 5.02	5.72	5.75 +/− 4.26	4.96
26	C	25	7.64 +/− 6.12	6.82	6.86 +/− 5.40	6.10

**Table 3:** Post-treatment abundance detected at the colony level (for details, see Table 2). As a treatment effect could be detected, the group means are shown also. Some AH colonies resulted as negative (i.e., zero *N. ceranae* copies/bee), which precluded the transformation into logarithms. Those cases are indicated with an asterisk and represent a non-significant (*16S rRNA* (χ^2^ (1, N = 19) = 2.287, *p* = 0.319)) and, respectively, significant (*Hsp70* (χ^2^ (1, N = 19) = 7.719, *p* = 0.021)) proportion of colonies that became negative.

			***16S rRNA***		***Hsp70***	
**Colony**	**Group**	**N**	**Mean +/− s.e.**	**SD**	**Mean +/− s.e.**	**SD**
3	AB	25	4.45 +/− 3.36	4.06	3.76 +/− 2.08	2.78
4	AB	25	3.54 +/− 2.68	3.38	2.77 +/− 1.01	1.71
5	AB	25	2.87 +/− 1.42	2.12	2.31 +/− 1.08	1.78
8	AB	25	2.86 +/− 1.45	2.15	2.24 +/− 1.11	1.81
10	AB	25	3.55 +/− 2.45	3.15	2.86 +/− 1.23	1.93
13	AB	25	3.53 +/− 2.50	3.20	2.86 +/− 1.35	2.04
14	AB	25	2.73 +/− 1.36	2.06	2.35 +/− 0.92	1.62
**Group AB**		**175**	**3.76 +/− 3.57**	**4.00**	**3.08 +/− 2.89**	**3.31**
F1	AH	25	2.85 +/− 1.11	1.81	2.51 +/− 1.64	2.34
F3	AH	25	2.86 +/− 1.07	1.77	2.47 +/− 1.39	2.09
16	AH	25	0.34 +/− 0.11	0.81	−*	−*
22	AH	25	2.87 +/− 1.14	1.84	2.25 +/− 1.16	1.86
24	AH	25	0.56 +/− 0.30	1.00	−*	−*
25	AH	25	−*	−*	−*	−*
**Group AH**		**150**	**2.56 +/− 2.21**	**2.60**	**2.12 +/− 1.80**	**2.19**
F4	C	25	5.56 +/− 4.37	5.07	4.93 +/− 3.06	3.76
F7	C	25	5.58 +/− 4.25	4.95	4.93 +/− 3.07	3.77
F9	C	25	4.90 +/− 3.45	4.15	4.00 +/− 2.18	2.88
17	C	25	5.46 +/− 4.07	4.77	4.89 +/− 3.03	3.73
19	C	25	6.84 +/− 5.28	5.98	5.81 +/− 4.06	4.76
26	C	25	8.69 +/− 7.33	8.03	6.92 +/− 5.49	6.19
**Group C**		**150**	**7.92 +/− 7.91**	**8.30**	**6.18 +/− 6.13**	**6.52**

**Table 4:** Post hoc test for the multiple comparison of the percent prevalence variations calculated in the treatment groups. The Bonferroni-corrected *p*-values referring to the *16S* *rRNA* and *Hsp70* methods are shown respectively above and below the diagonal.

	AB	AH	C
**AB**	-	0.051	1.000
**AH**	0.049	-	0.064
**C**	1.000	0.062	-

**Table 5:** Treatment effect on *N. ceranae* abundance.

	*16S rRNA*			*Hsp70*		
Group	Mean +/− s.e.	SD	95% CI	Mean +/− s.e.	SD	95% CI
AB	–99.38 +/− 0.28	0.75	−100.07, −98.69	–98.75 +/− 0.56	0.56	−100.12, −97.37
AH	–100.00 +/− 0.00	0.00	−100.00, −99.99	–99.98 +/− 0.01	0.03	−100.00, −99.94
C	+164.80 +/− 170.50	417.64	−273.49, +603.09	+40.52 +/− 12.98	31.80	−7.14, +73.89

**Table 6:** Characterization of the regression model relating the abundance ratio *16S rRNA*/*Hsp70* (dependent variable) to the *Hsp70* abundance (independent variable).

**Model**	F(1873) = 1627.70, *p* = 0.000, Adj. R^2^ = 0.650
**Intercept**	12.82 +/− 1.80 s.e. (95% CI = 9.28, 16.35), t(873) = 7.119, *p* = 0.000
**Slope**	6.68 +/− 0.16 s.e. (95% CI = 6.36, 7.00) × 10^−9^, t(873) = 40.345, *p* = 0.000
